# Overlap of Afferent Baroreflex Failure and Lower Cranial Neuropathies in Long-term Oropharyngeal Cancer Survivors Postradiation Therapy: Secondary Analysis of a Prospective Registry Cohort

**DOI:** 10.1016/j.adro.2026.102121

**Published:** 2026-06-20

**Authors:** Efthymios Triantafyllou, Isabelle Jang, Ariana J. Sahli, Patrik J. Van-Bergen, Amy C. Moreno, Anita Deswal, Elie Mouhayar, Clifton D. Fuller, Katherine A. Hutcheson, Efstratios Koutroumpakis

**Affiliations:** a Department of Cardiology, Division of Internal Medicine; b Department of Head and Neck Surgery, Division of Surgery; c Department of Radiation Oncology, The University of Texas MD Anderson Cancer Center, Houston, Texas

## Abstract

**Purpose:**

Afferent baroreflex failure (ABF) is an underrecognized late complication of radiation therapy (RT) in oropharyngeal cancer (OPC) survivors. Patients with ABF present with labile blood pressure, palpitations, lightheadedness, and syncope. ABF is attributed to fibrotic injury of the carotid sinus nerve and shares pathophysiology with lower cranial neuropathies (LCNP), which are routinely screened as part of head and neck physical examination in OPC survivorship. We evaluated the prevalence of ABF in patients with LCNPs and prior history of OPC treated with RT.

**Methods and Materials:**

This is a secondary analysis of a prospective cohort of OPC survivors treated with RT at a tertiary cancer center (2016-2019). ABF manifestations included new or worsening hypertension, hypotension requiring intervention, arrhythmias, or syncope. LCNP was defined as motor signs of neuropathy of the glossopharyngeal, vagus, or hypoglossal cranial nerves ≥3 months after therapy documented in the electronic health record.

**Results:**

Among 414 patients, 87% were men and 91% white with a median age of 60 (54-67) years at cancer diagnosis. During a median follow-up of 6.3 years (Interquartile range, 5.2-7), LCNP was diagnosed in 23 patients (5.8%). ABF manifestations were reported in 82 patients (19.6%) in the whole cohort (labile hypertension in 9.2%, hypotension in 7%, arrhythmias in 3.9%, syncope in 3.9%). ABF manifestations were present among 39% of patients with LCNP compared with 18.3% without LCNP (OR = 2.93; 95% CI, 1.22-7.06; *P* = .016).

**Conclusions:**

In this cohort of long-term OPC survivors treated with RT, over one-third of patients with LCNP had manifestations linked to ABF. A low threshold for dedicated ABF evaluation may be warranted in this high-risk subgroup.

## Introduction

Oropharyngeal cancer (OPC) is the most common subtype of head and neck cancer, accounting for approximately 60,000 new cases each year in the United States.^1^ It increasingly affects younger individuals (<55 years), which can be attributed to the rising prevalence of human papillomavirus infection.^2^ As survival rates improve, the focus has shifted from cancer treatment to include the management of therapy-related complications, especially the late effects of radiation therapy (RT). Afferent baroreflex failure (ABF) is a debilitating and underrecognized sequela of RT, manifesting as labile blood pressure, recurrent syncope, and falls several years after treatment.^3^ Lower cranial neuropathies (LCNP) represent another serious complication in OPC survivors treated with RT.^4^ Radiation damages the glossopharyngeal (IX), vagus (X), and hypoglossal (XII) cranial nerves, impairing swallowing, speech, and airway protection. Both complications share similar pathophysiology, as radiation induces injury and fibrosis of cranial nerve fibers and the carotid sinus.^5^^,^^6^ However, LCNPs are better documented than ABF, because cranial nerve function is routinely evaluated on head and neck examination in survivorship clinics. The aim of this study was to evaluate the prevalence of overlap syndrome between ABF-associated manifestations and other LCNPs in long-term (>5 years) OPC survivors treated with RT and to identify a potential subgroup suitable for targeted autonomic testing/surveillance.

## Methods and Materials

### Patient population

Patients diagnosed with oropharyngeal cancer and treated with RT at a tertiary cancer center between 2016 and 2019 were retrospectively identified. Baseline demographic and clinical characteristics, as well as ABF-related manifestations and LCNP diagnoses, were extracted from electronic medical records. The study was approved by the institutional review board, and the requirement for informed consent was waived.

### Clinical variable definition

To account for dosimetric and radiobiological differences in delivered treatment, carotid-specific iso-effective equivalent dose in 2 Gy fractions (EQD2) values were calculated using the linear-quadratic model applied to collected radiation dose data.^7^ ABF-related manifestations included new onset hypertension or worsening hypertension requiring titration of treatment, hypotension requiring pharmacological intervention, sustained arrhythmias, and syncope. LCNPs were diagnosed using published criteria at least 3 months after RT, excluding other sources of injury.^8^ CN IX and X neuropathies required absent pharyngeal constriction and abnormal vocal cord mobility, whereas CN XII required at least 2 of the following findings: tongue atrophy, deviation, or fasciculations.

### Endpoints and statistical analysis

We evaluated the frequency of overlap manifestations between LCNP and ABF following RT in patients with OPC. Additionally, we evaluated the association of baseline demographics, as well as cardiovascular and cancer-related clinical variables, with ABF-related manifestations and LCNP.

Categorical variables are described as frequencies (percentages), whereas continuous variables are summarized as mean ± SD or median [Interquartile Range(IQR)]. Chi-square was used to compare the frequency of ABF symptoms between patients with and without LCNPs, and logistic regression was used to identify significant associations with ABF symptoms as the endpoint. Two Fine-Gray competing risks regression models were constructed to identify predictors of ABF and LCNP, with death as the competing event and radiotherapy start as the index date. Variables with *P* < .10 in univariable analysis were entered into multivariable models.

## Results

A total of 414 OPC survivors were identified, of whom 24 lacked available data for LCNP screening. At the time of RT initiation, the median age was 60 (IQR, 54-67) years, 87.2% were men, and 90.8% were white. The mean body mass index was 28.8 (26-31.8) kg/m². Cardiovascular risk factors were documented at baseline in 88%; 51.7% had hypertension, 45.7% had dyslipidemia, 15.5% diabetes mellitus, and 53.4% used tobacco products. A history of established cardiovascular disease was present in 23.4%, with coronary artery disease accounting for 8%. Statins were prescribed for 35.1% and antiplatelet therapy for 27.8% of the population. Median EQD2 RT dose delivered was 70.7 (IQR, 68.6-71.6) Gy, with 86% receiving concurrent chemotherapy, 27.9% immunotherapy, and 11% undergoing surgery. Baseline characteristics are summarized in Table 1.

**Table 1:** Baseline demographic and clinical characteristics of patients with oropharyngeal cancer treated with RT

Covariate (N = 414)	N (%)
Age at RT (y), median (IQR)	60 (54-67)
Male sex	361 (87.2)
White race	376 (90.8)
Hispanic ethnicity	38 (9.2)
BMI (kg/m^2^), median (IQR)	28.8 (26-31.8)
History of CV risk factors	364 (88.1)
Obesity (BMI >30 kg/m^2^)	158 (38.5)
Tobacco use	221 (53.4)
Hypertension	214 (51.7)
Dyslipidemia	189 (45.7)
Diabetes mellitus	64 (15.5)
Prior history of cardiovascular disease	97 (23.4)
Coronary artery disease	33 (8)
Arrhythmias	32 (7.7)
CVE/TIA	19 (4.6)
Prior carotid stenosis >50%	18 (4.3)
Statins	145 (35.1)
Antiplatelet therapy	115 (27.8)
Chemotherapy	356 (86)
Platinum based	269 (65)
Taxane based	110 (26.6)
Immunotherapy	115 (27.9)
Surgery (N-missing = 21)	42 (10.7)
RT dose (Gy), median (IQR)	70.7 (68.6-71.6)

*Abbreviations:* BMI, body mass index; CV, cardiovascular; CVE, cerebrovascular events; IQR, interquartile range; RT, radiation therapy; TIA, transient ischemic attack

Over a median follow-up of 6.3 years (IQR, 5.2-7), LCNPs developed in 23 patients (5.8%), including 4 with CN IX involvement, 4 with CN X injury, and 18 with CN XII neuropathy. ABF-related manifestations occurred in 82 patients (19.6%), including new or worsened hypertension in 38 (9.2%), hypotension in 29 (7%), new arrhythmias in 16 (3.9%), and syncope in 16 patients (3.9%). All-cause mortality occurred in 24.4%.

When comparing patients with LCNP to those without, the prevalence of ABF-related manifestations was 39.1% versus 18% (*P* = .013). Patients with LCNPs were nearly 3 times more likely to exhibit ABF-related symptoms (OR = 2.93; 95% CI, 1.22-7.06; *P* = .016). Among patients with LCNP, the most common ABF-related manifestation was new or worsened hypertension requiring medication in 6 patients, followed by syncope in 4 patients. No other significant differences were observed between the 2 groups.

Older age (sHR, 1.03; 95% CI, 1.01-1.06; *P* = .006) and higher EQD2 RT dose (sHR, 1.01; 95% CI, 1.00-1.02; *P* = .007) were independently associated with ABF-related manifestations (Supplementary Table E1), whereas only taxane-based chemotherapy (sHR, 2.41; 95% CI, 1.02-5.73; *P* = .046) was independently associated with LCNP (Supplementary Table E2), in the multivariable Fine-Gray models accounting for death as a competing risk.

## Discussion

To our knowledge, this is the first study to examine the prevalence of overlap syndrome between LCNP and ABF-related manifestations after RT in a cohort of long-term survivors of OPC. In this retrospective study, we found that over one-third of patients with LCNP had manifestations related to ABF, with a threefold increased risk of concurrent ABF-related manifestations. These findings support the hypothesis that the coexistence of these 2 radiation-related complications identifies a subpopulation in which targeted autonomic testing for ABF diagnosis could be considered.

The carotid baroreceptor is innervated by branches of the CN IX and X.^3^ Cranial nerves IX and X provide the afferent pathway, sensing changes in blood pressure, whereas efferent fibers mediate reflex cardiovascular adjustments to maintain blood pressure within a normal range. Radiation causes carotid sinus injury and cranial nerve fibrosis that eventually leads to the development of baroreflex failure and LCNPs, respectively.^5^^,^^9^ Other factors described in the literature that potentially increase the risk of ABF development are surgery and platinum- and taxane-based chemotherapy.^10^ In our study, older age at RT and higher EQD2 RT dose were associated with ABF-related manifestations, whereas surgery and chemotherapy did not. This discrepancy may be explained by the limited number of patients who underwent surgery alone or who did not receive chemotherapy in our cohort, which reduced our statistical power to detect significant associations for these variables. Additionally, we found that patients with LCNPs have a higher risk of developing ABF-related symptoms, which can be supported by the common underlying pathophysiology. Because OPC survivors often get screened for cranial neuropathies in the survivorship clinics, early dysfunction in any of the lower cranial nerves should raise concern for the subclinical baroreflex dysfunction.

Afferent baroreflex failure develops several years after RT with debilitating symptoms.^10^ The diagnosis of ABF is made by assessing the autonomic nervous system—including both the adrenergic and cardiovagal components. The test evaluates blood pressure and heart rate responses during the Valsalva maneuver, deep breathing, tilt table, and cold pressor tests. Additionally, plasma norepinephrine levels have been proposed to assist with diagnosis.^11^ Currently, there are no screening protocols for ABF in patients with a history of head and neck irradiation and the diagnosis is often delayed, losing an opportunity to explore preventive therapies. The goal of this work is to raise awareness that ABF-related manifestations, which are nonspecific, are common among head and neck cancer survivors and that providers should have a low threshold for referring these patients for dedicated autonomic testing.

Our work is subject to limitations. First, this study is retrospective and carries the risk of selection bias. The presence of ABF is based on the presence of manifestations related to the disease that are nonspecific. The median follow-up of 6 years may underestimate the true incidence of ABF, as many cases develop beyond that time period. Standardized autonomic testing was not performed. Additionally, we did not perform carotid and carotid sinus dosimetry, precluding a more mechanistically precise assessment of the dose-response relationship between radiation, ABF-associated symptoms, and LCNP. Nevertheless, this is, to our knowledge, the first study to examine the overlap between LCNP and ABF after RT and paves the way for larger studies, which may identify the benefit of early screening in this subgroup and allow the exploration of preventive therapies.

In summary, over one-third of patients with LCNPs and a prior history of neck irradiation for OPC also had ABF-related manifestations. A low threshold for dedicated ABF evaluation using autonomic testing may be warranted in this high-risk group.

## Disclosures

E.K. has received grant support from NIH and Cancer Prevention and Research Institute of Texas and consulting fees from Sumitomo. A.D. has received consultancy fees from Bayer. C.D.F. reports unrelated grant support from the National Institutes of Health, NRG Oncology, and Elekta AB; unrelated honoraria/meals/travel/consulting relationships from NIH, NRG Oncology, Elekta AB, Siemens Healthineers/Varian Medical Systems, Philips Medical Systems, and GE Healthcare; and unrelated royalties from Kalliso, Inc. All other authors declare no competing interests.

## Appendix Supplementary materials

Supplemental material Image, application 1

## Footnotes

Supplementary material associated with this article can be found in the online version at doi:10.1016/j.adro.2026.102121.
