# Supplementary material for: Overlap of Afferent Baroreflex Failure and Lower Cranial Neuropathies in Long-term Oropharyngeal Cancer Survivors Postradiation Therapy: Secondary Analysis of a Prospective Registry Cohort
Source: Adv Radiat Oncol. 2026 Jun 20;11(11):102121. doi: 10.1016/j.adro.2026.102121 (PMC13400387; doi:10.1016/j.adro.2026.102121)
Supplement: Supplemental material [file mmc1.docx]

| **Supplemental Table 1. Univariable and multivariable Fine-Gray analysis with ABF-related manifestations as an endpoint** | | | | |
| --- | --- | --- | --- | --- |
| Covariate (N=414) | **Univariable SHR (95% CI), p** | **Multivariable SHR (95% CI), p** |  |  |
| Age at RT (years), mean (SD) | 1.03 (1.01–1.05), p = 0.007 | 1.03 (1.01–1.06), p = 0.006 |  |  |
| Male sex | 1.37 (0.67–2.82), p = 0.390 | - |  |  |
| BMI (kg/ m^2^), mean (SD) | 1.02 (0.97–1.08), p = 0.450 | - |  |  |
| Obesity (BMI>30 kg/m^2^) | 0.84 (0.51–1.40), p = 0.510 | - |  |  |
| Smoking | 0.89 (0.58–1.39), p = 0.620 |  |  |  |
| Hypertension | 1.43 (0.92–2.24), p = 0.110 | - |  |  |
| Dyslipidemia | 1.38 (0.89–2.14), p = 0.150 | - |  |  |
| Diabetes | 0.83 (0.42–1.62), p = 0.580 | - |  |  |
| Prior History of Cardiovascular disease | 1.00 (0.59–1.70), p = 0.990 | - |  |  |
| Statins | 1.17 (0.74–1.82), p = 0.500 | - |  |  |
| Antiplatelet therapy | 1.26 (0.79–2.01), p = 0.330 | - |  |  |
| Chemotherapy |  |  |  |  |
| Platinum based | 1.25 (0.79–1.99), p = 0.340 | - |  |  |
| Taxane based | 1.40 (0.87–2.26), p = 0.170 | - |  |  |
| Surgery | 1.08 (0.54–2.15), p = 0.820 | - |  |  |
| Surgery + Chemotherapy | 0.82 (0.31–2.22), p = 0.700 | - |  |  |
| EQD2 RT dose (Gy), mean (SD) | 1.01 (1.00–1.02), p = 0.053 | 1.01 (1.00–1.02), p = 0.007 |  |  |
| ABF- Afferent Baroreflex Failure, BMI-Body Mass Index, RT-Radiation Therapy | | |  |  |

| **Supplemental Table 2. Univariable and multivariable Fine-Gray analysis with LCNP as an endpoint** | | | | |
| --- | --- | --- | --- | --- |
| Covariate (N=414) | **Univariable SHR (95% CI), p** | **Multivariable SHR (95% CI), p** |  |  |
| Age at RT (years), mean (SD) | 1.01 (0.96–1.06), p = 0.690 | — |  |  |
| Male sex | 1.32 (0.30–5.72), p = 0.710 | — |  |  |
| BMI (kg/ m^2^), mean (SD) | 0.95 (0.85–1.07), p = 0.400 | - |  |  |
| Obesity (BMI>30 kg/m^2^) | 0.72 (0.27–1.94), p = 0.520 | - |  |  |
| Smoking | 0.96 (0.42–2.23), p = 0.930 |  |  |  |
| Hypertension | 0.57 (0.25–1.33), p = 0.200 | - |  |  |
| Dyslipidemia | 0.87 (0.37–2.02), p = 0.740 | - |  |  |
| Diabetes | 0.55 (0.13–2.33), p = 0.420 | - |  |  |
| Prior History of Cardiovascular disease | 1.29 (0.50–3.28), p = 0.600 | - |  |  |
| Statins | 0.70 (0.27–1.77), p = 0.450 | - |  |  |
| Antiplatelet therapy | 0.65 (0.25–1.73), p = 0.390 | - |  |  |
| Chemotherapy |  |  |  |  |
| Platinum based | 1.69 (0.68–4.20), p = 0.260 | - |  |  |
| Taxane based | 2.21 (0.95–5.13), p = 0.066 | 2.41 (1.02–5.73), p = 0.046 |  |  |
| Surgery | 0.91 (0.21–3.84), p = 0.900 | - |  |  |
| Surgery + Chemotherapy | 1.68 (0.40–6.99), p = 0.480 | - |  |  |
| EQD2 RT dose (Gy), mean (SD) | 1.01 (1.00–1.03), p = 0.064 | 1.01 (1.00–1.03), p = 0.051 |  |  |
| BMI-Body Mass Index, LCNP- Lower Cranial Neuropathies, RT-Radiation Therapy | | |  |  |
